# Temporal expression study of miRNAs in the crown tissues of winter wheat grown under natural growth conditions

**DOI:** 10.1186/s12864-021-08048-5

**Published:** 2021-11-04

**Authors:** Menglei Wang, Chenhui Yang, Kangning Wei, Miao Zhao, Liqiang Shen, Jie Ji, Li Wang, Daijing Zhang, Junqiang Guo, Yun Zheng, Juanjuan Yu, Mo Zhu, Haiying Liu, Yong-Fang Li

**Affiliations:** 1 College of Life Sciences, Henan Normal University, Xinxiang, 453007 Henan China; 2 Henan International Joint Laboratory of Agricultural Microbial Ecology and Technology, Henan Normal University, Xinxiang, 453007 China; 3 Present address: National Engineering Laboratory for Tree Breeding, College of Biological Sciences and Technology, Beijing Forestry University, Beijing, 100083 China; 4 Jindal School of Management, University of Texas at Dallas, 800 W Campbell RD, Richardson, TX 75080 USA; 5 Faculty of Information Engineering and Automation, Kunming University of Science and Technology, Kunming, 650500 Yunnan China; 6 State Key Laboratory of Primate Biomedical Research, Institute of Primate Translational Medicine, Kunming University of Science and Technology, Kunming, 650500 Yunnan China

**Keywords:** Winter wheat, miRNA, Crown, Target gene, Cold acclimation, Post-transcriptional regulation

## Abstract

**Background:**

Winter wheat requires prolonged exposure to low temperature to initiate flowering (vernalization). Shoot apical meristem of the crown is the site of cold perception, which produces leaf primordia during vegetative growth before developing into floral primordia at the initiation of the reproductive phase. Although many essential genes for winter wheat cold acclimation and floral initiation have been revealed, the importance of microRNA (miRNA) meditated post-transcriptional regulation in crowns is not well understood. To understand the potential roles of miRNAs in crown tissues, we performed a temporal expression study of miRNAs in crown tissues at the three-leaf stage, winter dormancy stage, spring green-up stage, and jointing stage of winter wheat grown under natural growth conditions.

**Results:**

In total, 348 miRNAs belonging to 298 miRNA families, were identified in wheat crown tissues. Among them, 92 differentially expressed miRNAs (DEMs) were found to be significantly regulated from the three-leaf stage to the jointing stage. Most of these DEMs were highly expressed at the three-leaf stage and winter dormancy stage, and then declined in later stages. Six DEMs, including miR156a-5p were markedly induced during the winter dormancy stage. Eleven DEMs, including miR159a.1, miR390a-5p, miR393-5p, miR160a-5p, and miR1436, were highly expressed at the green-up stage. Twelve DEMs, such as miR172a-5p, miR394a, miR319b-3p, and miR9676-5p were highly induced at the jointing stage. Moreover, 14 novel target genes of nine wheat or *Pooideae*-specific miRNAs were verified using RLM-5′ RACE assay. Notably, six *mTERF*s and two *Rf1* genes, which are associated with mitochondrial gene expression, were confirmed as targets of three wheat-specific miRNAs.

**Conclusions:**

The present study not only confirmed the known miRNAs associated with phase transition and floral development, but also identified a number of wheat or *Pooideae*-specific miRNAs critical for winter wheat cold acclimation and floral development. Most importantly, this study provided experimental evidence that miRNA could regulate mitochondrial gene expression by targeting *mTERF* and *Rf1* genes. Our study provides valuable information for further exploration of the mechanism of miRNA mediated post-transcriptional regulation during winter wheat vernalization and inflorescent initiation.

**Supplementary Information:**

The online version contains supplementary material available at 10.1186/s12864-021-08048-5.

## Background

MicroRNA (miRNA) is a kind of small, single strand non-coding RNA regulating target gene expression at the post-transcriptional level. miRNAs regulate target genes through sequence complementary based cleavage or translation repression [1–4]. The crucial roles of miRNAs in a number of processes, for example, plant growth, developmental, and stress responsive, have been proven by the pleiotropic developmental defects observed in *dcl1*, *hyl1*, *hen1*, *se1*, and *ago1* mutants [3]. Most conserved miRNAs target transcription factors (TFs), which can either activate or suppress the expression of downstream genes [5]. The miR156 and miR172 regulation loops that control plant vegetative/reproductive phase transition have been shown to be highly conserved among phylogenetically distinct plant species [6–11]. miR156 targets squamosa promoter binding like (SPL) TFs, while, miR172 targets the APETALA2 (AP2) family TFs, a class of flowering suppressor genes [12, 13]. miR156 is highly expressed in the early growth stage and then declines with plant age, while the expression of miR172 is significantly induced at the reproductive stage. The age-dependent decline of miR156 expression level is associated with a subsequent increase in *SPL* expression, which, in turn, promotes the expression of miR172 and initiates floral induction [8, 10, 11]. miR159 targets *GAMYB* TFs that are involved in the gibberellin (GA) flowering pathway. Previous studies on wheat, barley, and rice have confirmed that miR159 is associated with anther development [14–16]. miR399 is induced under phosphate starvation conditions, which helps to improve phosphate uptake by downregulating the expression level of the *PHOSPHATE 2 (PHO2)* gene. Recent studies have shown that the accumulation of miR399 is also influenced by ambient temperature [17]. Both miR399 overexpression and loss-of-function *PHO2* mutants showed early flowering phenotypes in Arabidopsis grown at 23 °C, indicating that miR399 is also associated with flowering time control [18].

Wheat (*Triticum aestivum* L.) is an important cereal crop globally. Winter wheat must undergo a certain term of cold temperature to initiate flowering. Studies of grafting and localized cooling have shown that the shoot apical meristem (SAM) of the crown tissue is the site of cold perception during vernalization [19]. The SAM of wheat produces leaf primordia during vegetative growth and then develops into floral primordia at the initiation of the reproductive phase. Several crucial vernalization associated genes, such as *VRN1*, *VRN2*, *VRN3*, *VER2* (encoding a carbohydrate-binding protein), and wheat *GRP2*, an RNA-binding protein gene, have been confirmed play essential roles during wheat transition from vegetative growth to reproductive stage [20–24].

Wheat miRNAs have been widely characterized using high-throughput sequencing technique [25–31]; miRNAs related to wheat seed development [25, 26, 32–34] and cold stress response [35, 36] have been studied. Two miRNAs had been confirmed closely associated with wheat flowering through regulating two key vernalization associated gene families. *FLOWERING LOCUS T (FT)* is a key gene regulating Arabidopsis flowering time. FT is a mobile florigen, expressed in leaves and then transported to SAM through the phloem, where it initiates phase transition [37]. Similar to FT, VRN3 is an important flowering promoter in wheat. Expression of *VRN3* is suppressed under short-day conditions, and can be activated by vernalization [24]. *VRN3* is targeted by miR5200 through cleavage mechanism in wheat and *Brachypodium distachyon* [38, 39], where miR5200 was highly expressed in the leaves under short-day, but was dramatically suppressed under long-day conditions, which is opposite to the expression of *VRN3* [39]. As a key member of the plant circadian clock, *TIME OF CAB EXPRESSION 1* (*TOC1*) is closely related to the CO-FT flowering pathway [40]. Beyond of plantacyanin and copper-containing protein genes, wheat *TOC1s* have been confirmed as targets of miR408, a copper deprivation responsive miRNA [5, 41, 42]. Further study revealed that the suppression of *TOC1s* by miR408 could activate the expression of *VRN3* and stimulate wheat phase transition [42]. We previously studied the miRNA profiles in the leaves of winter wheat grown under field conditions that consisted of various environmental factors [43]. However, less information is available on miRNA mediated post-transcriptional regulation in crown tissues during the process of cold acclimation and floral initiation. Due to the important characters that SAM is the cold preceptor and produces leaf / floral primordia at different growth stages, we performed a genome-wide study of miRNAs in the wheat crown tissues at the three-leaf stage, winter dormancy stage, spring green-up stage, and jointing stage, aiming to understand the roles of miRNAs during winter wheat cold acclimation and phase transition.

## Results

### Overview of small RNA (sRNA) sequencing data

**Fig. 1:**
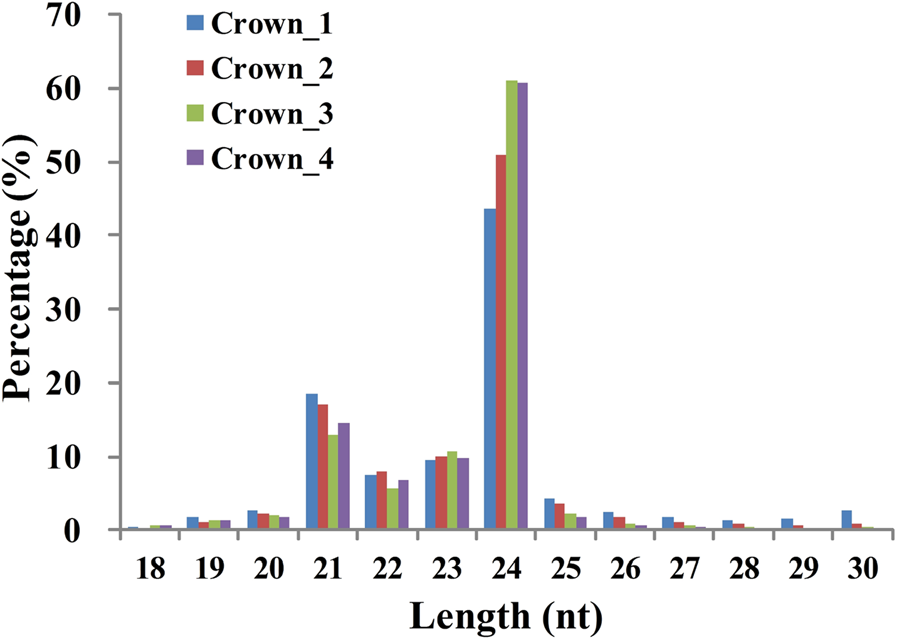
Size distribution of sRNAs in wheat crown tissues at four different growth and development stages. Crown_1, Crown_2, Crown_3, and Crown _4 represent the three-leaf stage, winter dormancy stage, spring green-up stage, and jointing stage, respectively

To investigate the temporal expression of miRNAs in the crown tissues of winter wheat grown under field conditions, four sRNA libraries (Crown_1, Crown_2, Crown_3 and Crown_4) were constructed and deep sequenced from crown tissues at the three-leaf stage, winter dormancy stage, spring green-up stage and jointing stage, respectively. Approximately, 24 to 26 million raw reads of 18–30 nt in length were obtained for each library. The size distribution of sRNAs in crowns were similar to that in wheat leaves and other studies [43], with fragment sizes mainly at 21 nt and 24 nt (Fig. 1). After removing the low-quality reads, adaptor sequences, reads less than 18 nt, and poly(A) reads, a total of 23.4 to 25.2 million clean reads per library were obtained, which represented 11,340,259 to 14,182,109 unique reads; these data reflect the highly diverse and complex sRNA population expressed in wheat crown tissues. The overall ratio of clean reads and unique reads mapped to the wheat genome were 87.65 and 84.69%, respectively (Table 1), indicating the high quality of the sRNA libraries.

**Table 1:** Summary of 4 small RNA libraries from wheat crown tissues

Sample	Raw reads	Clean reads	Mapped reads	rRNA	snRNA	snoRNA	tRNA	Unique reads	Unique mapped reads
Crown_1	26,128,505	25,255,156 (100%)	21,674,636 (85.82%)	1,684,229 (6.67%)	5410 (0.02%)	2664 (0.01%)	175,006 (0.69%)	11,555,577 (100%)	9,645,563 (83.47%)
Crown_2	24,410,817	23,676,703 (100%)	20,533,438 (86.87%)	1,092,515 (4.61%)	4720 (0.02%)	2311 (0.01%)	134,203 (0.57%)	11,340,259 (100%)	9,308,344 (82.08%)
Crown_3	25,662,925	24,521,540 (100%)	21,888,604 (89.26%)	865,231 (3.57%)	4851 (0.02%)	2228 (0.01%)	81,307 (0.33%)	14,182,109 (100%)	12,230,315 (86.24%)
Crown_4	24,347,926	23,408,612 (100%)	20,800,808 (88.86%)	672,087 (2.87%)	6783 (0.03%)	1433 (0.01%)	118,341 (0.51%)	13,526,123 (100%)	11,671,922 (86.29%)
Total	100,550,173	96,862,011 (100%)	84,897,486 (87.65%)	4,314,062 (4.45%)	21,764 (0.02%)	8636 (0.01%)	508,857 (0.53%)	50,604,068 (100%)	42,856,144 (84.69%)

Note: Crown_1 to Crown_4 represent sRNA libraries generated from crown tissues collected at the three-leaf stage, winter dormancy stage, spring green-up stage and jointing stage, respectively

### Identification of miRNAs expressed in wheat crown tissues

To identify the known miRNAs expressed in wheat crown tissues, the unique reads were first aligned against all mature plant miRNAs deposited in the miRBase database (Release 22.1). In total, 282 known miRNAs corresponding to 233 miRNA families were identified (Additional file 1). There were 224, 227, 227, and 213 miRNAs found at the three-leaf stage, winter dormancy stage, spring green-up stage, and jointing stage, respectively. A total of 165 miRNAs were detected at all four stages, 72 miRNAs were detected at more than two stages, and 45 miRNAs were only expressed at one stage (Additional file 2). These results indicated that most of the known miRNAs were expressed throughout the entire growth period in this study, and only a small number of miRNAs were specific to a certain developmental stage. With the exception of miR162, 21 highly conserved plant miRNA families (miR156/157, miR159, miR160, miR164, miR165/166, miR167, miR168, miR169, miR170/171, miR172, miR319, miR390, miR391, miR393, miR394, miR395, miR396, miR397, miR398, miR399, and miR408), and the conserved monocot-specific miR444 were found in crown tissues. The expression of conserved miRNAs was generally higher than that of non-conserved miRNAs. The miR159 family topped the list of conserved miRNAs, followed by miR168, miR171, miR319, miR165, miR164, miR167, miR396, miR160, and miR444. The expression of different members of the same miRNA family was very different (Additional file 1). Among the non-conserved miRNAs, the expression of miR5048, miR5062, miR7757, miR9652, miR9662, miR9674, miR9772, and miR9773 were very high in all of the four growth stages; miR5240, miR6249, and miR9672 were highly expressed in at least one stage.

**Fig. 2:**
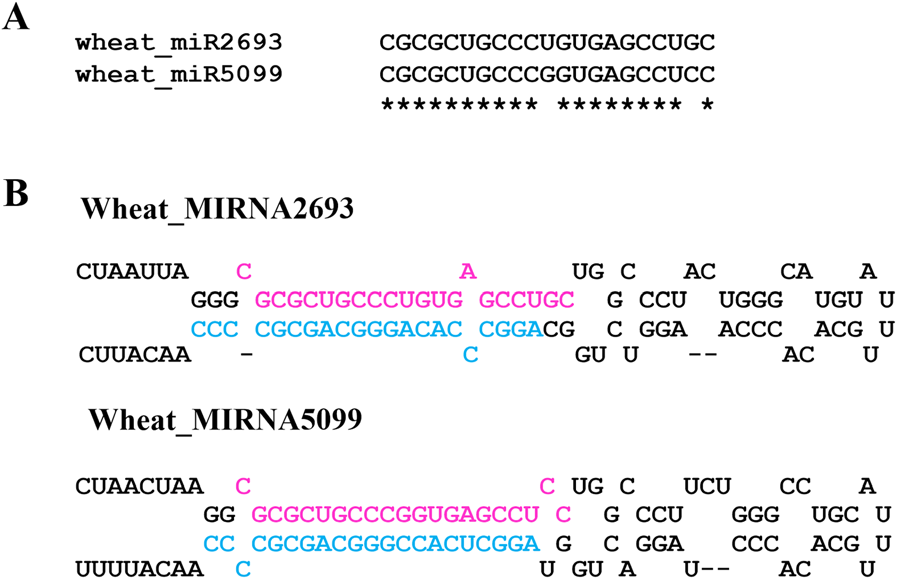
Two new wheat miRNAs identified in crown tissues. **A** Sequence alignment of wheat_miR2693 and wheat_miR5099. Asterisk represents the same nucleotides between wheat_miR2693 and wheat_miR5099. **B** The 5′ to 3′ secondary structure of the precursors of wheat_miR2693 and wheat_miR5099 predicted by Mfold. The mature miRNA and miRNA* are indicated in pink and blue color, respectively

We reported 55 novel miRNAs and 27 putative novel miRNAs in winter wheat leaf tissues [43], among them, 41 novel miRNAs and 23 putative miRNAs were detected in wheat crown tissues too, confirming the reliability of these sRNAs as miRNAs; these miRNAs were named wheat_miRNAs in this study (Additional file 3). Moreover, two new miRNAs (wheat_miR2693 and wheat_miR5099) with detectable miRNA* sequences were identified in crown tissues. These two miRNAs belonged to a miRNA family with 2 nt variation (Fig. 2). Further analysis showed that they were homologous to wheat novel miRNA3036a-1 and miRNA3036a-2 [44], but with different genomic location. Information on these two new miRNAs is shown in Additional file 4: Table S3. Totally, 348 miRNAs belonging to 298 miRNA families were detected in wheat crown tissues.

### Differentially expressed miRNAs (DEMs) in wheat crown tissues during wheat growth

**Fig. 3:**
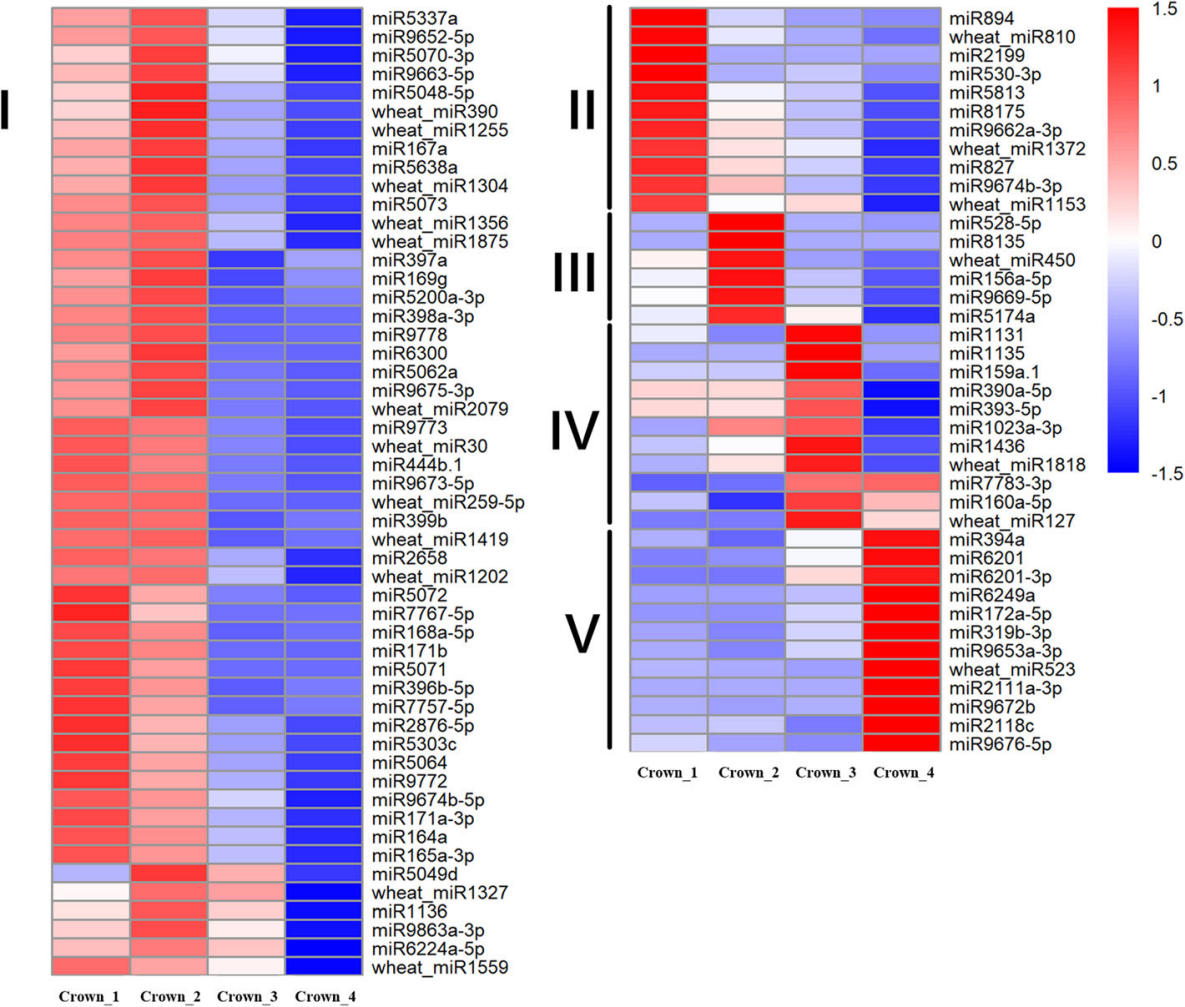
Heat map of differentially expressed miRNAs in wheat crown tissues at four different stages. I to V represent 5 clusters with different expression pattern. The bar represents the scale of relative expression levels of miRNAs. The expression of miRNAs were normalized by Z-score normalization

**Fig. 4:**
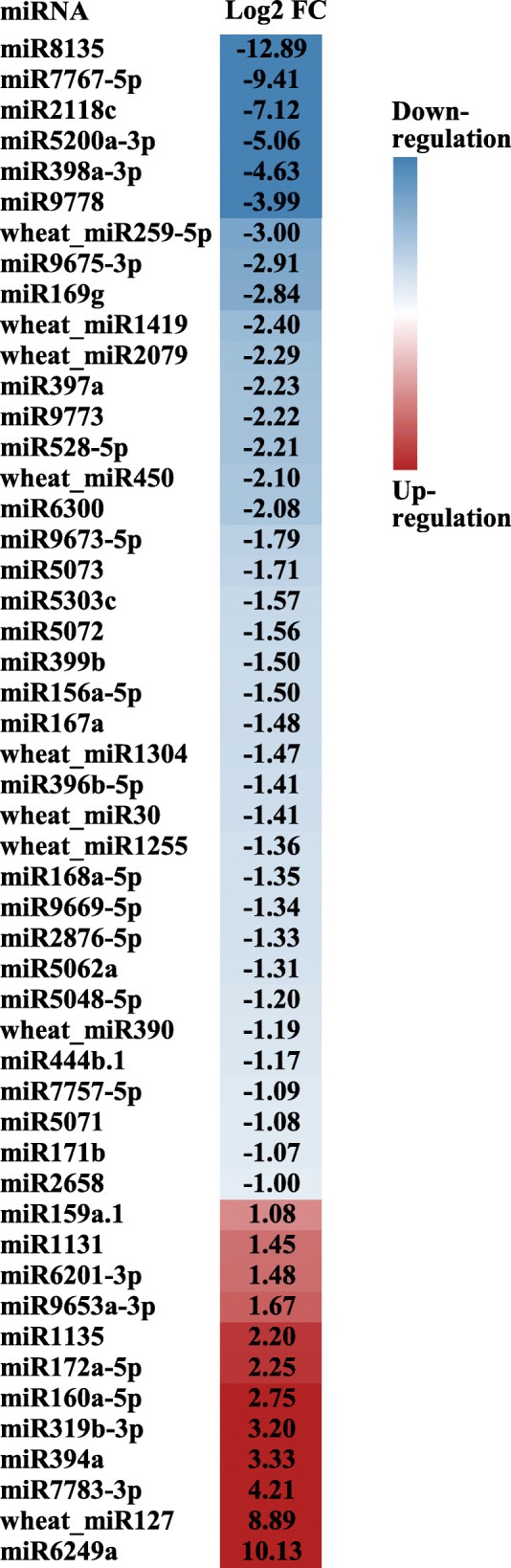
Expression of miRNAs significantly regulated in crown tissues during the transition from the winter dormancy stage to the spring green-up stage

Comparison of miRNA expression between different growth stages could provide insights into the post-transcriptional mechanism mediated by miRNAs during winter wheat cold acclimation and flowering initiation. In total, 92 miRNAs were significantly regulated in wheat crown tissues from the three-leaf stage to the jointing stage (Additional file 5), including 19 miRNAs that were first reported in wheat leaves [43]. Based on the expression patterns, these DEMs were grouped into five clusters. The first cluster consisted of 52 miRNAs that were highly expressed at the three-leaf stage and the winter dormancy stage. Most of these DEMs were suppressed at the spring green-up stage and the jointing stage, with the exception of six miRNAs (miR5049d, miR1136, miR9863a-3p, miR6224a-5p, wheat_miR1327, and wheat_miR1559), which were still highly expressed at the green-up stage (Fig. 3). Some of these miRNAs, such as miR5072, miR171b, miR164a, and miR165a-3p, showed the highest expression levels at the three-leaf stage, while others, such as miR5337a, miR9652-5p, miR167a, miR169g, miR397a, and wheat_miR1356, were highly expressed during the winter dormancy stage, miR444b.1, miR399b, and wheat_miR1202 showed the similar expression levels at both of the three-leaf stage and winter dormancy stage. There were 11 DEMs in cluster II, which showed the highest expression at the three-leaf stage, then declined with winter wheat growth. In cluster III, six DEMs, including miR156a-5p, were highly induced at the winter dormancy stage. In cluster IV, 11 DEMs, including miR159a.1, miR390a-5p, miR393-5p, miR160a-5p and miR1436, were highly induced at the green-up stage. Cluster V consisted of 12 DEMs, including miR394a, miR172a-5p, miR319b-3p, and miR9676-5p, that were significantly induced at the jointing stage (Fig. 3). We further checked the miRNA expression changes from the winter dormancy stage to the spring green-up stage, 50 DEMs (38 downregulated, 12 upregulated) were significantly regulated. As expected, miR156 and miR172 were included, as well as miR160, miR167, miR169, miR319, miR394, and miR444 (Fig. 4). Surprisingly, many of these DEMs were wheat or *Pooideae-*specific miRNAs. Together, these DEMs implied the complicated post-transcriptional regulation in crown tissues during wheat growth and development.

**Fig. 5:**
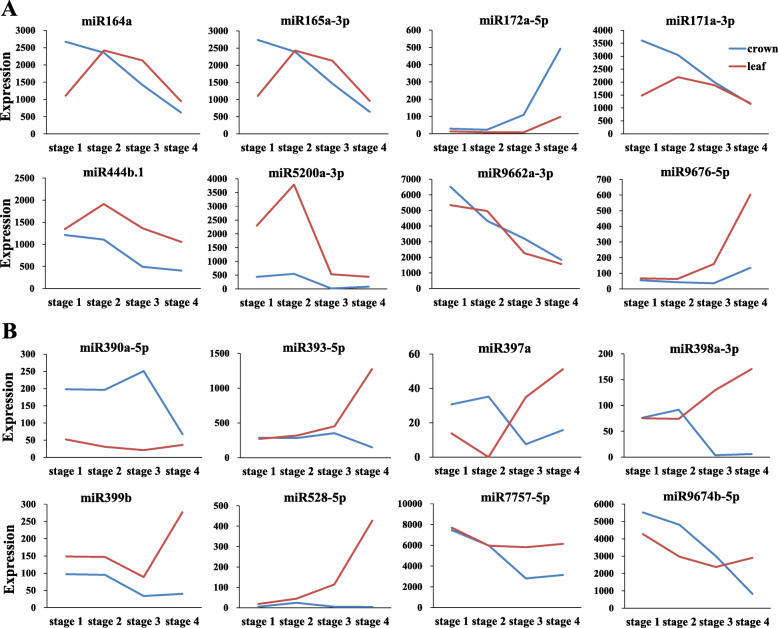
Expression trend comparison of DEMs in crown and leaf tissues during wheat development. **A** similar expression patterns. **B** different expression patterns

We further compared the expression pattern of DEMs in crown and leaf tissues [43] at the winter dormancy stage, spring green-up stage, and jointing stage, especially those miRNAs with identified target genes. Some DEMs, including miR156, miR164a, miR165a-3p, miR171a-3p, miR172a-5p, miR444b.1, miR5048-5p, miR5174a, miR9662a-3p, miR5072, and wheat_miR1356 displayed similar expression patterns in the two tissues (Fig. 5A & Additional file 6), while others, such as miR390a-5p, miR393-5p, miR397a, miR398a-3p, miR528-5p, miR167a, 168a-5p, miR169g, miR319b-3p, miR396b-5p, and miR7757-5p displayed different, even opposite expression patterns (Fig. 5B & Additional file 7). miR397a, miR398a-3p, and miR167a were suppressed in crowns at the green-up and jointing stages, however, they were induced in leaves. Although some miRNAs showed similar expression trends in leaves and in crowns, abundance differences were obvious at the two tissues. miR172a-5p was more abundant in crowns than in leaves, while miR5200a-3p and miR444 b.1 were more abundant in leaves than in crowns (Fig. 5).

**Fig. 6:**
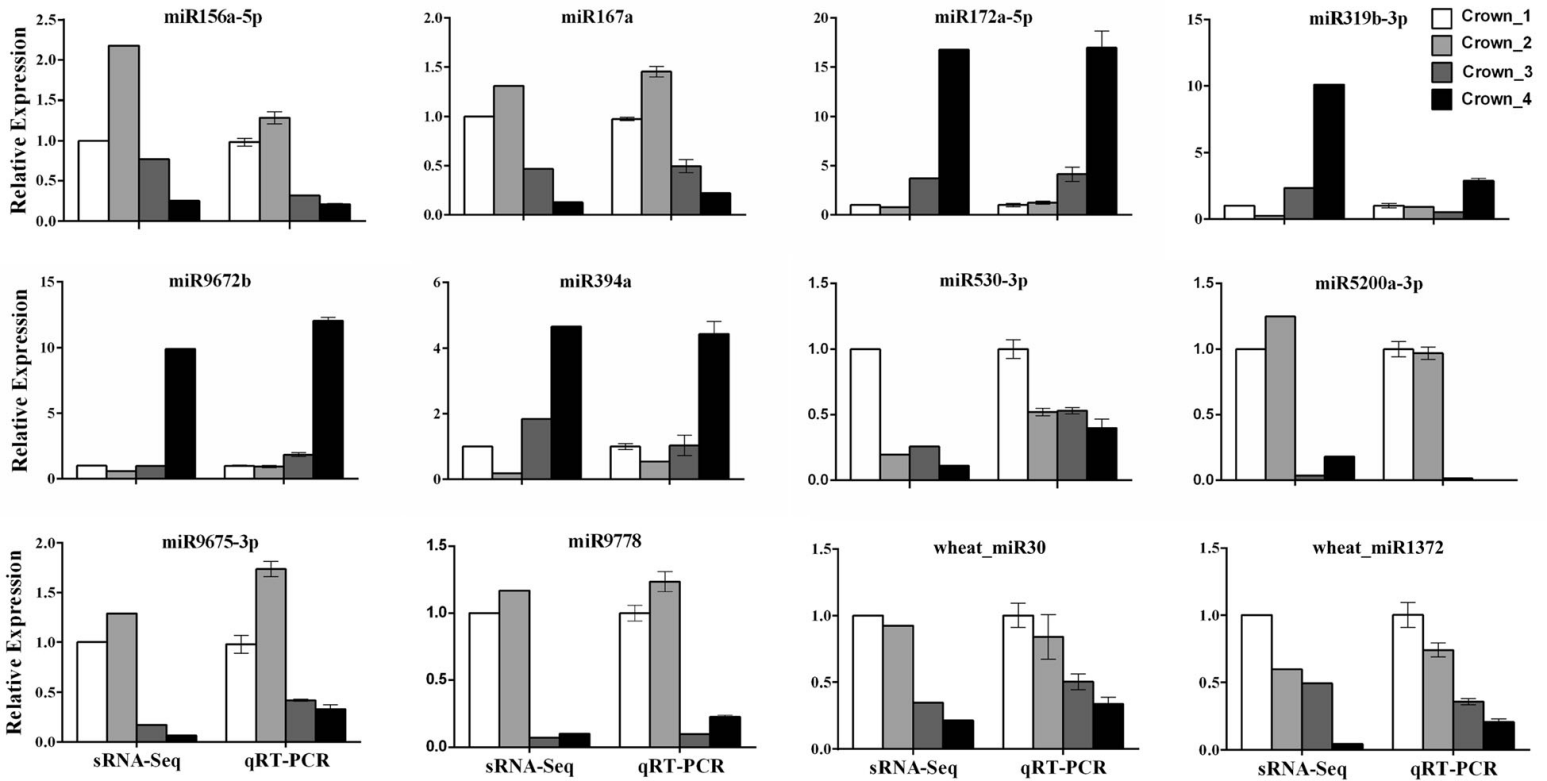
Quantitative RT-PCR analysis of differentially expressed miRNAs derived from sRNA-Seq. U6 snRNA was used as a reference. The error bars represent the standard deviation (SD) of three replicates

To validate the reliability of the sRNA-Seq data, we randomly selected 14 DEMs for quantitative reverse transcription PCR (qRT-PCR) analysis. The expression patterns of 12 miRNAs from both the qRT-PCR and the deep sequencing data were similar, despite some quantitative differences in expression levels (Fig. 6), indicating that the sRNA sequencing results obtained in this study were reliable.

### Validation of wheat or *pooideae*-specific miRNA targets

**Fig. 7:**
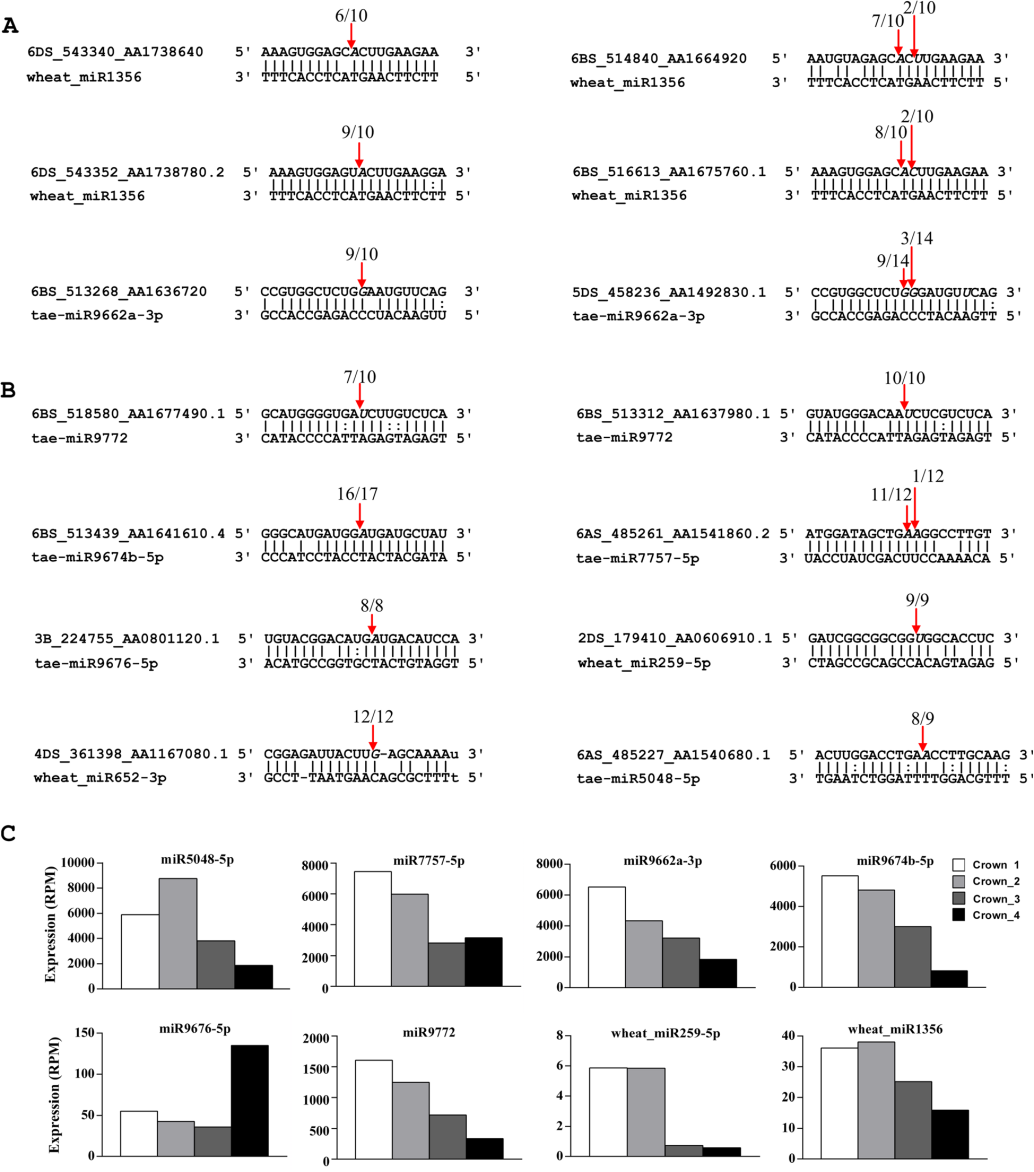
Validation of 14 novel targets for 9 miRNAs using RLM-5′ RACE assay. Red arrows represent the detected cleavage sites. Gene ID, 6BS _516613_AA1675760.1, is the abbreviation of TRIAE_CS42_6BS_TGACv1_516613_AA1675760.1. **A** mitochondrial transcription termination factors (mTERFs) targeted by miR9662a-3p and wheat_miR1356. **B** Other novel targets of non-conserved miRNAs. **C** Expression of the eight DEMs in crown tissues

To illustrate the potential function of the non-conserved wheat or *pooideae*-specific miRNAs, we used RLM-5′ RACE assay to validate some of the predicted target genes [43]. In total, 14 novel target genes for nine DEMs were verified (Table 2, Fig. 7). miR9662a-3p, a wheat-specific miRNA, has been shown to target TRIAE_CS42_6DS_TGACv1_542718_AA1728780, a mitochondrial transcription termination factor (mTERF) or mitochondrial transcription factor [43]. In this study, two other *mTERF*s, TRIAE_CS42_6BS_TGACv1_513268_AA1636720 and TRIAE_CS42_5DS_TGACv1_458236_AA1492830, were confirmed as targets of miR9662a-3p (Fig. 7A). Wheat_miR1356 was first identified in winter wheat leaves [43], four *mTERF* genes were verified as *bona fide* targets of wheat_miR1356 in this study (Fig. 7A). mTERFs have recently emerged as central players in mitochondrial gene expression in various eukaryotes [45], our results indicated that miRNA could potentially affect plant organellar gene expression by regulating the expression of *mTERF* genes. We verified that miR5048, found in wheat and barley, targeted a putative terpene synthase gene. miR7757, reported in wheat and *B. distachyon*, was confirmed to target a disease resistance protein RGA5-like gene. miR9674 and miR9772 are found in wheat and its ancestor *Aegilops tauschii,* TRIAE_CS42_6BS_TGACv1_513439_AA1641610 (protein Rf1) and two F-box genes were confirmed as their corresponding targets. miR9676 is a wheat-specific miRNA, it targets TRIAE_CS42_3B_TGACv1_224755_AA0801120, a biostress-resistance-related protein. In addition, TRIAE_CS42_2DS_TGACv1_179410_AA0606910 (a kinesin-like gene) and TRIAE_CS42_4DS_TGACv1_361398_AA1167080 (a FAF-like) were validated as the targets of wheat_miR259-5p and wheat_miR652-3p, respectively (Fig. 7B). Among the nine DEMs, wheat_miR652-3p was expressed at low levels across all four stages (Additional file 3), miR9676-5p was highly induced at the jointing stage, the remaining seven miRNAs were highly expressed in the vegetative stages and suppressed in the transition and floral development stages (Fig. 7C). These data indicated that these wheat or *pooideae*-specific miRNAs were functional in regulating wheat growth and development through post-transcriptional cleavages of their target genes.

**Table 2:** Fourteen novel targets validated by RLM-5′ RACE assay for nine wheat or *pooideae* specific miRNAs

miRNA	miRNA conservation	Target gene ID	Target gene annotation
wheat_miR1356	tae	TRIAE_CS42_6BS_TGACv1_516613_AA1675760.1	mTERF, mitochondrial transcription factor
		TRIAE_CS42_6DS_TGACv1_543352_AA1738780.2	mTERF, mitochondrial transcription factor
		TRIAE_CS42_6DS_TGACv1_543340_AA1738640.3	mTERF, mitochondrial transcription factor
		TRIAE_CS42_6BS_TGACv1_514840_AA1664920.1	mTERF, mitochondrial transcription factor
miR9662a-3p	tae	TRIAE_CS42_5DS_TGACv1_458236_AA1492830.1	mTERF, mitochondrial transcription factor
		TRIAE_CS42_6BS_TGACv1_513268_AA1636720.1	mTERF, mitochondrial transcription factor
miR9772	tae, ata	TRIAE_CS42_6BS_TGACv1_513312_AA1637980.1	F-box domain containing protein
		TRIAE_CS42_6BS_TGACv1_518580_AA1677490.1	F-box domain containing protein
miR9674b-5p	tae, ata	TRIAE_CS42_6BS_TGACv1_513439_AA1641610.4	Protein Rf1. PPR repeat domain containing protein
miR7757-5p	tae, bdi	TRIAE_CS42_6AS_TGACv1_485261_AA1541860.2	disease resistance protein RGA5-like
miR9676-5p	tae	TRIAE_CS42_3B_TGACv1_224755_AA0801120.1	biostress-resistance-related protein
wheat_miR259-5p	tae	TRIAE_CS42_2DS_TGACv1_179410_AA0606910.1	Kinesin-like protein KIF15
wheat_miR652-3p	tae	TRIAE_CS42_4DS_TGACv1_361398_AA1167080.1	protein FAF-like, chloroplastic
miR5048-5p	tae, hvu	TRIAE_CS42_6AS_TGACv1_485227_AA1540680.1	putative terpene synthase

Note: tae, ata, bdi and hvu design *Triticum aestivum, Aegilops tauschii*, *Brachypodium distachyon* and *Hordeum vulgare*, respectively

## Discussion

The initiation of flowering in plants is regulated by complex gene networks that are influenced by environmental and endogenous cues to ensure that plants flower at the favorable time and environmental conditions. Under natural growth conditions, winter wheat must undergo a long period of low temperature in winter, and then transit from vegetative growth to reproductive growth in next spring. During this period, wheat must adjust its growth and development in response to changes in ambient temperature, light, and other environmental factors. The SAM of the crown tissue is the essential site of cold perception, which produces leaf primordia or floral primordia at the vegetative growth and reproductive phase, respectively. Therefore, we performed a transcriptome study of miRNAs expressed in wheat crown tissues at the three-leaf stage, winter dormancy stage, spring green-up stage, and jointing stage. In total, 348 miRNAs belonging to 298 miRNA families were identified in this study. With the exception of miR162, all of the most conserved miRNAs across the plant kingdom were found. The miR159 and miR168 families showed markedly high expression levels in crown tissues, which was similar to related findings in wheat leaves and rice [43, 46]. Non-conserved miRNAs, such as miR5048, miR5062, miR7757, miR9652, miR9662, miR9674, miR9772, and miR9773, were also highly expressed in the crown tissues across the studied stages. Moreover, 64 novel miRNAs that have been reported in wheat leaves [43] were also found in this study, confirming the reliability of these sRNAs as miRNAs.

The close association between miRNA and stress is revealed by the fact that miR398 targets Cu/Zn superoxide dismutases (*Cu/Zn SODs*) and a copper chaperone for Cu/Zn SOD (*CCS1*) [5, 47]. Strong upregulation of miR393 and downregulation of miR319 and miR398 were observed in Arabidopsis under cold stress [48]. Many conserved miRNAs and non-conserved miRNAs are found cold stress responsive in diverse plant species [49]. miR319b-3p was suppressed in crown tissues during the winter dormancy stage (Additional file 1 & Additional file 7), which was in accordance with the findings in Arabidopsis and wheat leaves [43, 48], while, most previously reported cold responsive miRNAs showed similar expression levels at both the three-leaf stage and winter dormancy stage (Fig. 3, Additional file 1 & Additional file 3). This was ascribed to the fact that most previous studies were conducted in controlled temperature environments for short term cold stress, while the samples used in the present study were collected from winter wheat grown in the field under natural weather conditions; thus, the wheat was not under cold stress, but went through cold acclimation [43].

Several miRNAs that are important for Arabidopsis and other plant vegetative/reproductive phase transition were found to be regulated in winter wheat. For example, miR156a-5p was highly expressed in the winter dormancy stage and declined at the green-up and jointing stages, while the expression of miR172a-5p was very low at the vegetative stage, then markedly induced at the green-up stage and reached its highest expression level at the jointing stage (Fig. 6). This finding indicates that the miR156 and miR172 regulation loop is conserved in wheat and other plant species. miR172 was more induced in the crown tissues than that in the leaves (Fig. 5), which ties well with the function of *AP2*, target of miR172, as flower repressors [12, 13]. miR319 targets *TCP* TFs, which play major roles in different developmental processes, including flower production, gametophyte, and leaf development [50]. miR319b-3p was constitutively induced at the green-up stage and jointing stage (Fig. 3 and Additional file 5), indicating that miR319 might be essential in regulating SAM differentiation into leaf meristem or floral meristem. Lower expression of miR399b in wheat crowns was detected at the green-up and jointing stages than at the vegetative growth stages, which confirms the findings that miR399 is a temperature-responsive miRNA [17], and the miR399-*PHO2* module might have roles in regulating flowering time in wheat as in Arabidopsis [18]. Expression of miR5200a at the green-up and jointing stages were lower than that at the vegetative stages (Fig. 6 and Additional file 5), which is consistent with its expression in leaves of *B. distachyon* and wheat [39, 43], and agrees well with the finding that *VRN3*, target of miR5200, is significantly induced at the reproductive stage [24]. In addition, the expression of miR5200a was dramatically higher in leaves than in crowns during winter dormancy stage (Fig. 5A), which could suppress the expression of *VRN3* and prevent wheat flowering at cold winter.

Plant hormone is one of the main factors affecting flowering initiation and development. miR159 targets *MYB* TFs that are implicated in the GA flowering pathway. Expression of miR159a.1 was suppressed in leaves at the spring green-up and jointing stages [43], while it was induced in crowns at the spring green-up stage, then markedly declined at jointing stage (Fig. S3), which is similar to its expression pattern observed in Arabidopsis [51], and is consistent with previous findings that miR159 is a flowering suppressor [16]. Several miRNAs target auxin response factors (ARFs) that further affect plant growth and phase transition. miR390 affects not only root development and leaf morphogenesis, but also influences the flowering process by prolonging the juvenile phase of Arabidopsis [52, 53]. miR390 triggers the production of ta-siRNA3 from the *TAS3* locus, and ta-siRNA3 negatively regulate the expression of *ARF3* and *ARF4* [54]. Both miR160 and miR167 also target different *ARF* members [5]. Wheat miR390 was suppressed at the jointing stage, the expression of miR167a declined at the spring green-up stage and reached its lowest expression level at the jointing stage, indicating that miR390 and miR167a are negative regulators during the winter wheat juvenile-adult phase transition, which is similar to that in Arabidopsis [52, 55]. Loss-of-function of ath-miR160a leading to irregular flowers, reduced fertility and aberrant seeds in Arabidopsis [56]. In this study, expression of miR160a-5p was markedly induced at the green-up stage, then slightly decreased at jointing stage, together with the fact that miR160a-5p was more abundantly expressed in the crowns than in the leaves (Additional file 6), we speculate that miR160 could regulate flowering by targeting *ARFs* in wheat.

Many species-specific miRNAs have emerged during plant evolution. A big portion of DEMs identified in this study were non-conserved, wheat or *Pooideae*-specific miRNAs. Target identification is the premises to determine whether these miRNAs are functional and what are their functions. Using RLM-5′ RACE assay, 14 genes were verified as novel targets of nine wheat or *Pooideae*-specific miRNAs (Table 2). Mitochondria are critical for energy production and cell signaling. Mitochondria have their own genome (mitochondrial DNA, mtDNA), RNA, and ribosomes; therefore, the regulation of mtDNA expression is critical for meeting energy demands during particular growth and developmental stages, or in response to environmental changes [57]. The majority of mTERFs in plants are predicted to be plastid- or mitochondria-localized, and have emerged as central players in mitochondrial gene expression in various eukaryotes [45]. Four *mTERF*s were confirmed as targets of wheat_miR1356, and two other *mTERF*s were found to be experimentally cleaved by miR9662a-3p (Fig. 7A). We previously verified that TRIAE_CS42_6DS_TGACv1_542718_AA1728780 (*mTERF*) is a target of miR9662a-3p [43]. In total, seven *mTERFs* were confirmed as targets of miRNAs in wheat. Pentatricopeptide repeat (PPR)-containing proteins are one of the largest protein families in plants, and half of the PPR proteins function within mitochondria [58]. Cytoplasmic male sterility (CMS) is a maternally inherited feature that prevents the production of functional pollen. Several PPR proteins have been proven to be CMS fertility restorers in different species [59–63]. Genetic and biochemical analyses have shown that PPR proteins can affect RNA processing with their PPR motif in a sequence-specific manner [58, 64, 65]. Two *Rf1* genes (PPR proteins), TRIAE_CS42_6BS_TGACv1_513439_AA1641660, validated previously [43], and TRIAE_CS42_6BS_TGACv1_513439_AA1641610 (Fig. 7B), were verified as targets of miR9674b-5p in wheat. The expression of miR9662a-3p, wheat_miR1356, and miR9674b-5p were high at the three-leaf stage and winter dormancy stage, and gradually decreased with the growth of winter wheat plants (Figs. 3, 7C & Additional file 5). Therefore, we speculate that these three miRNAs are essential for winter wheat growth and development by targeting *mTERF* and *Rf1* genes, which, in turn, regulate mitochondrial gene expression, maturation, and stability.

Terpenoids, a kind of secondary metabolite, play important roles in plant defense and interactions with unfavorable environments. Plants have two different terpenoid biosynthesis pathways: the mevalonate (MVA) pathway and non-mevalonate, or methylerythritol 4-phosphate (MEP) pathway, which take place in the cytoplasm and plastid, respectively [66]. Several miRNAs, including miR396, miR398, miR530, miR6300, and miR6173, have been predicted to be involved in the terpene pathway due to the fact that their targets are the upstream genes in the MVA or MEP pathways [66, 67]. In this study, miR396, miR398, miR6300, and miR530 were induced/ suppressed during the winter dormancy stage. Moreover, we verified that miR5048-5p, which was highly expressed during the winter dormancy stage, targeted TRIAE_CS42_6AS_TGACv1_485227_AA1540680 (a putative terpene synthase) by post- transcriptional cleavage (Fig. 7B), which provided confirmative evidence that miRNA is directly associated with terpenoid biosynthesis, which might be beneficial for wheat survival in cold winter weather conditions.

Wheat_miR259-5p was first identified in wheat leaves [43]. TRIAE_CS42_2DS_TGACv1_179410_AA0606910, a kinesin-like protein of *KIF15*, was validated as a target of wheat_miR259-5p (Fig. 7B). Kinesin proteins are important microtubule-based motor proteins that play critical roles in mitosis, morphogenesis, and signal transduction. Plant kinesins are directly or indirectly associated with cell division and growth [68, 69]. Rice mutants of *bc12* (a kinesin-like protein, Brittle Culm12) displays a dwarfism phenotype [70]. Further study showed that BC12 possesses transcription regulation activity and mediates cell elongation by regulating the GA biosynthesis pathway in rice [71]. In this study, wheat_miR259-5p was highly expressed during vegetative growth and decreased at the spring green-up and jointing stages (Fig. 7C), indicating that *KIF15* might be suppressed in winter and induced in the warm spring season. This is correlated well with the low and sometimes ceased growth of wheat in the cold winter, and the fast growth at the green-up and jointing stages.

It is known that *FANTASTIC FOUR* (*FAF*) genes in Arabidopsis can regulate shoot meristem size [72]. We confirmed that wheat_miR652 targeted TRIAE_CS42_4DS_TGACv1_361398_AA1167080.1, a FAF-like gene (Fig. 7B). TRIAE_CS42_6AS_TGACv1_485261_AA1541860.2, disease resistance protein RGA5-like, was confirmed as a target of miR7757-5p (Fig. 7B), which was highly expressed during the winter dormancy stage, implying that miR7757-5p might play a role in the resistance of wheat to the cold weather in winter. TRIAE_CS42_3B_TGACv1_224755_AA0801120, a biostress-resistance-related gene, was proven as a target of miR9676-5p, which was significantly induced at the jointing stage. Two F-box domain containing protein genes were validated as targets of miR9772, which was highly expressed in the vegetative stages and decreased at the green-up and jointing stages (Figs. 6 & 7). Target validation is the basis for illustrating the function of miRNAs, further functional analysis of these target genes is required to determine the detailed roles of the corresponding miRNAs in wheat.

**Fig. 8:**
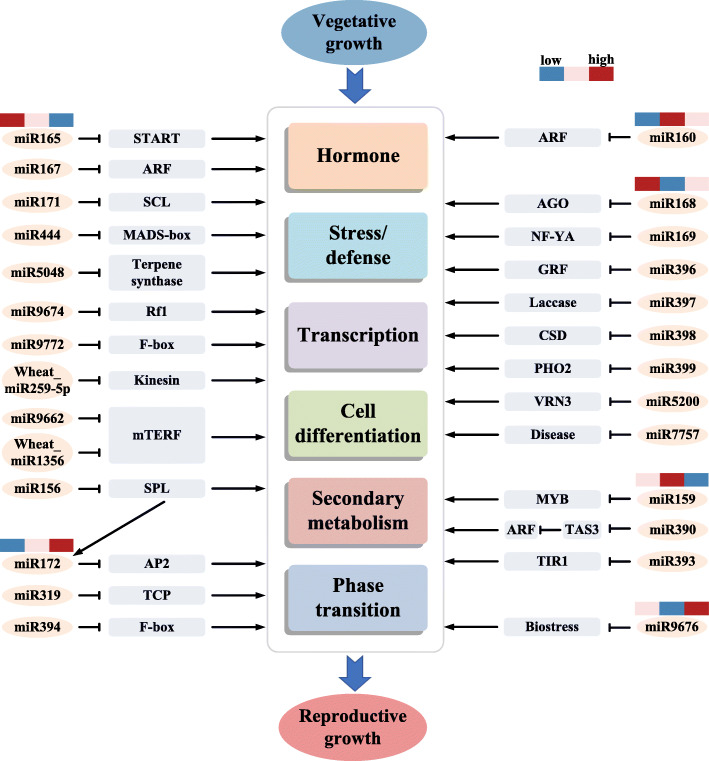
Schematic illustration of miRNA regulatory network in crown tissues associated with winter wheat transition from vegetative growth to reproductive growth. Letters inside square box indicate the miRNA target gene function. Color bar indicates miRNA expression pattern at winter dormancy stage, spring green-up stage and jointing stage. TF: transcription factor; AGO: Argonaute; AP2: Apetala 2; ARF: Auxin response factor; CSD: Copper/zinc superoxide dismutase; GRF: Growth regulating factor protein; mTERF: mitochondrial transcription termination factors; MYB: Myb-like DNA-binding domain containing protein; NF_YA: Nuclear transcription factor Y subunit alpha; PHO2: PHOSPHATE 2; Rf1: Release factors 1; SCL: Scarecrow-like protein; SPL: Squamosa promoter-binding like protein; START: START domain containing protein; TAS3: trans-acting small interfering RNA3; TCP: TEOSINTE-BRANCHED1/CYCLOIDEA/PCF; TIR: Transport inhibitor response 1 protein; VRN3: Vernalization 3

Target genes of conserved miRNAs are relatively studied well, and are conserved in all or most plant species [5, 27, 32, 43]. Based on the DEMs expression pattern at the later three stages, we constructed a model to show the DEMs and their corresponding targets regulation network during winter wheat transition from vegetative growth to reproductive growth (Fig. 8). Further studies to identify the target genes regulated by the non-conserved miRNAs will enrich this network.

## Conclusions

In total, 348 miRNAs belonging to 298 miRNA families were identified in wheat crown tissues, and 92 DEMs were significantly regulated from the three-leaf stage to jointing stage. Moreover, 14 novel target genes for nine wheat or *Pooideae*-specific miRNAs were verified using RLM-5′ RACE assay. The present study not only confirmed the known miRNAs associated with plant phase transition and floral development, but also identified a number of wheat or *Pooideae*-specific miRNAs critical for winter wheat growth and development. This study also provided experimental evidence that miRNA could regulate mitochondrial gene expression by targeting *mTERF* and *Rf1* genes. Our study provides valuable information for further exploration of the mechanism behind miRNA mediated post-transcriptional regulation during winter wheat vernalization and inflorescent initiation.

## Materials and methods

### Plant materials and growth

Shimai 22, a semi-winter wheat cultivar [73], was used in this study. Seeds were sewn in autumn. Crown tissues were collected at four different stages, that is, the three-leaf stage (Crown_1), winter dormancy stage (Crown_2), spring green-up stage or double ridge stage (Crown_3), and jointing stage (Crown_4). Three biological replicates were collected for each stage. All of the samples were collected between 1:30 and 3:00 PM [43]. All samples were frozen immediately in liquid nitrogen and stored at − 80 °C for total RNA isolation.

### Small RNA library construction and sequencing

Total RNA was extracted using TRIzol reagent (Thermo Fisher Scientific, USA). RNA quality and concentration were measured using 1% agarose gel electrophoresis and Nanodrop2000 measurements. Total RNA from the three biological replicates for each growth stage were equally pooled for sRNA library preparation. In total, four small RNA libraries (Crown_1, Crown_2, Crown_3, and Crown_4) were constructed in the following steps: sRNAs (18–30 nt) were purified using 15% polyacrylamide gel electrophoresis (PAGE), 3′ and 5′ RNA adaptor ligation, reverse transcribed into cDNA, library enrichment by PCR amplification, and sRNA library purification using PAGE [7, 43]. The quality and concentration of the sRNA libraries were checked using Agilent bioanalyzer 2100 and by quantitative PCR. The sRNA libraries that were barcoded for each sample were multiplexed and sequenced on a BGISEQ500RS sequencing platform (BGI, Shenzhen, China).

### Small RNA sequencing data analysis

Raw reads from high-throughput sequencing was filtered by removing low quality reads and trimming the adaptor sequences to obtain clean reads ranging from 18 nt to 30 nt. Reads derived from rRNAs, tRNAs, small nuclear RNAs (snRNAs), small nucleolar RNAs (snoRNAs), and repeat RNAs were further discarded using SOAP [27, 43, 74–76]. Identical reads were pooled to generate unique reads and corresponding frequencies. All mature plant miRNAs deposited in miRBase Release 22.1 (http://www.mirbase.org) were downloaded, and the redundant sequences were combined to obtain known plant miRNA sequences. Clean reads were used to identify known miRNAs expressed in crown tissues via BLASTN search against known miRNA sequences. Those with fewer than two mismatches with known plant miRNA sequences were designated as known wheat miRNAs; sRNA sequences were also searched by alignment with the novel miRNAs identified in wheat leaves in our previous study [43]. The remaining sequences were mapped to the wheat genome for novel miRNA identification using MIREAP software (https://sourceforge.net/projects/mireap/). The flanking genome sequences (from 100 nt upstream to 100 nt downstream) of the sRNAs were used to predict the secondary hairpin structures using Mfold [77]. Only those with more than 50 reads in at least two samples and detected miRNA* were retained, and the miRNA candidates were further screened by BLASTN against wheat miRNAs that were reported in the literature but not deposited in miRbase to determine whether they were novel miRNAs.

### Screening and validation of differentially expressed miRNAs

To identify the miRNAs that were differentially expressed in crown tissues during the four different stages, expression of miRNAs were normalized using Tags Per Million reads (TPM). The DEGseq pipeline [78] was used to identify DEMs using the following criteria: |log_2_ fold change| **>** 1, *p* < 0.01, with more than 5 TPM in at least one stage for each comparison. The miRNAs that met these criteria were designed as DEMs.

Expression pattern of DEMs were checked by qRT-PCR. Total RNA was first polyadenylated and then reverse transcribed into cDNA using a poly (T)-adaptor oligonucleotide according to the instruction of Mir-X miRNA qRT-PCR SYBR Kit (TaKaRa, Cat. No. 638313, Tokyo, Japan). miRNA sequences were used for miRNA specific forward primer designing. The cDNAs were diluted 10-fold for qRT-PCR analysis [43]. Dissociation curves were checked to exclude nonspecific amplifications. All reactions were repeated three times per sample. U6 snRNA was used as reference to normalize the expression of miRNAs. miRNA expression at the three-leaf stage (Crown_1) was set to 1.0, the relative expression of miRNA in other three stages were determined using the comparative threshold cycle (2^-ΔΔCT^) method [79]. The sequence of the all primers used in this study is presented in Additional file 8.

### Validation of miRNA target genes using RLM-5′ RACE assay

RNA ligase-mediated 5′-rapid amplification of cDNA ends (RLM-5′ RACE) was used to validate the potential target genes of the identified DEMs. A 5′ RNA adaptor (5′ GUU CAG AGU UCU ACAG UCC GAC 3′) was ligated to 5′end of RNA, oligo dT (18) primer and Superscript II reverse transcriptase (Thermo Fisher Scientific, USA) were used to synthesize cDNA. Nested PCR was performed and separated on a 2% agarose gel. Bands with expected sizes were purified and ligated to pMD19 T-vector (TaKaRa). Following transformation and colony PCR, plasmids were isolated from positive colonies and subjected to Sanger sequencing [5, 43].

## Supplementary Information

**Additional file 1: Table S1.** Expression of known miRNAs identified in wheat crown tissues.

**Additional file 2: Figure S1.** Common and specific known miRNAs expressed in wheat crown tissues at four different growth and development stages.

**Additional file 3: Table S2.** Expression of wheat new miRNAs in crown tissues.

**Additional file 4: Table S3.** Information of wheat_miRNA2693 and wheat_miR5099.

**Additional file 5: Table S4.** Expression of differentially expressed miRNAs in wheat crown tissues.

**Additional file 6: Figure S2.** miRNAs with similar expression patterns in crown and leaf tissues at the winter dormancy stage, spring green-up stage and jointing stage.

**Additional file 7: Figure S3.** miRNAs with different expression patterns in crown and leaf tissues at the winter dormancy stage, spring green-up stage and jointing stage.

**Additional file 8: Table S5.** Sequence of primers used in this study.

## Data Availability

The sRNA-Seq data obtained in this study was deposited at the National Center for Biotechnology Information Gene Expression Omnibus (NCBI, GEO, http://www.ncbi.nlm.gov.geo/) under accession number GSE155963.

## Abbreviations

miRNA: MicroRNA
sRNA: Small RNA
snRNAs: Small nuclear RNAs
snoRNAs: Small nucleolar RNAs
mtDNA: Mitochondrial DNA
TFs: Transcription factors
SPL: Squamosa promoter binding like
AP2: APETALA2
PHO2: PHOSPHATE 2
SAM: Shoot apical meristem
FT: FLOWERING LOCUS T
TOC1: TIME OF CAB EXPRESSION 1
DEMs: Differentially expressed miRNAs
mTERF: Mitochondrial transcription termination factor
SODs: Superoxide dismutases
ARFs: Auxin response factors
PPR: Pentatricopeptide repeat
CMS: Cytoplasmic male sterility
MVA: The mevalonate
MEP: Methylerythritol 4-phosphate
FAF: FANTASTIC FOUR
PAGE: Polyacrylamide gel electrophoresis
TPM: Tags Per Million
RLM-5′ RACE: RNA ligase-mediated 5′-rapid amplification of cDNA ends
SD: Standard deviation
