# Supplementary figures and images for: Temporal expression study of miRNAs in the crown tissues of winter wheat grown under natural growth conditions
Source: BMC Genomics. 2021 Nov 4;22(Suppl 3):793. doi: 10.1186/s12864-021-08048-5 (PMC8567549; doi:10.1186/s12864-021-08048-5)

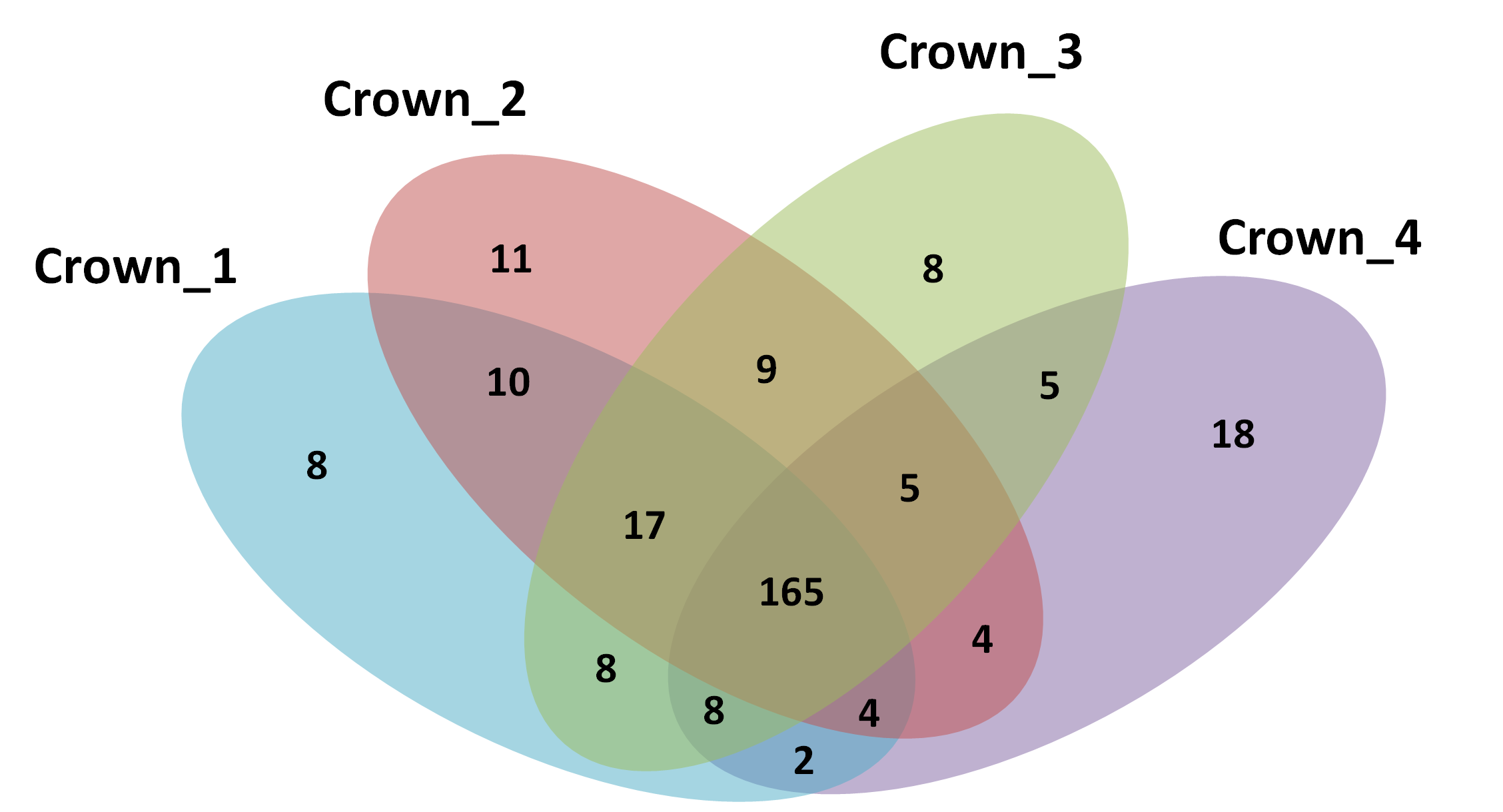

Supplement: Supplementary file 2 — Additional file 2: Figure S1. Common and specific known miRNAs expressed in wheat crown tissues at four different growth and development stages. [file 12864_2021_8048_MOESM2_ESM.tif]

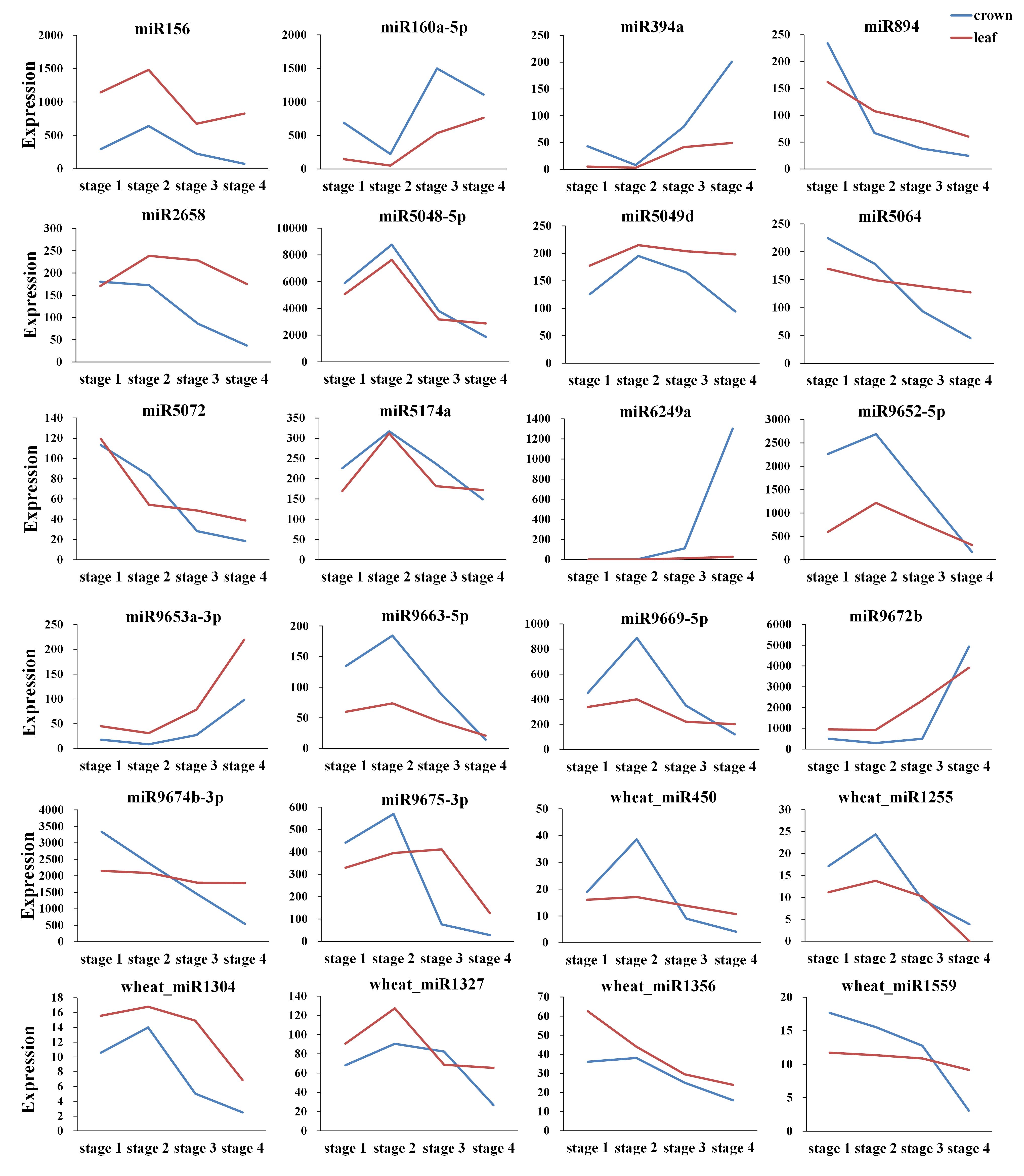

Supplement: Supplementary file 6 — Additional file 6: Figure S2. miRNAs with similar expression patterns in crown and leaf tissues at the winter dormancy stage, spring green-up stage and jointing stage. [file 12864_2021_8048_MOESM6_ESM.jpg]

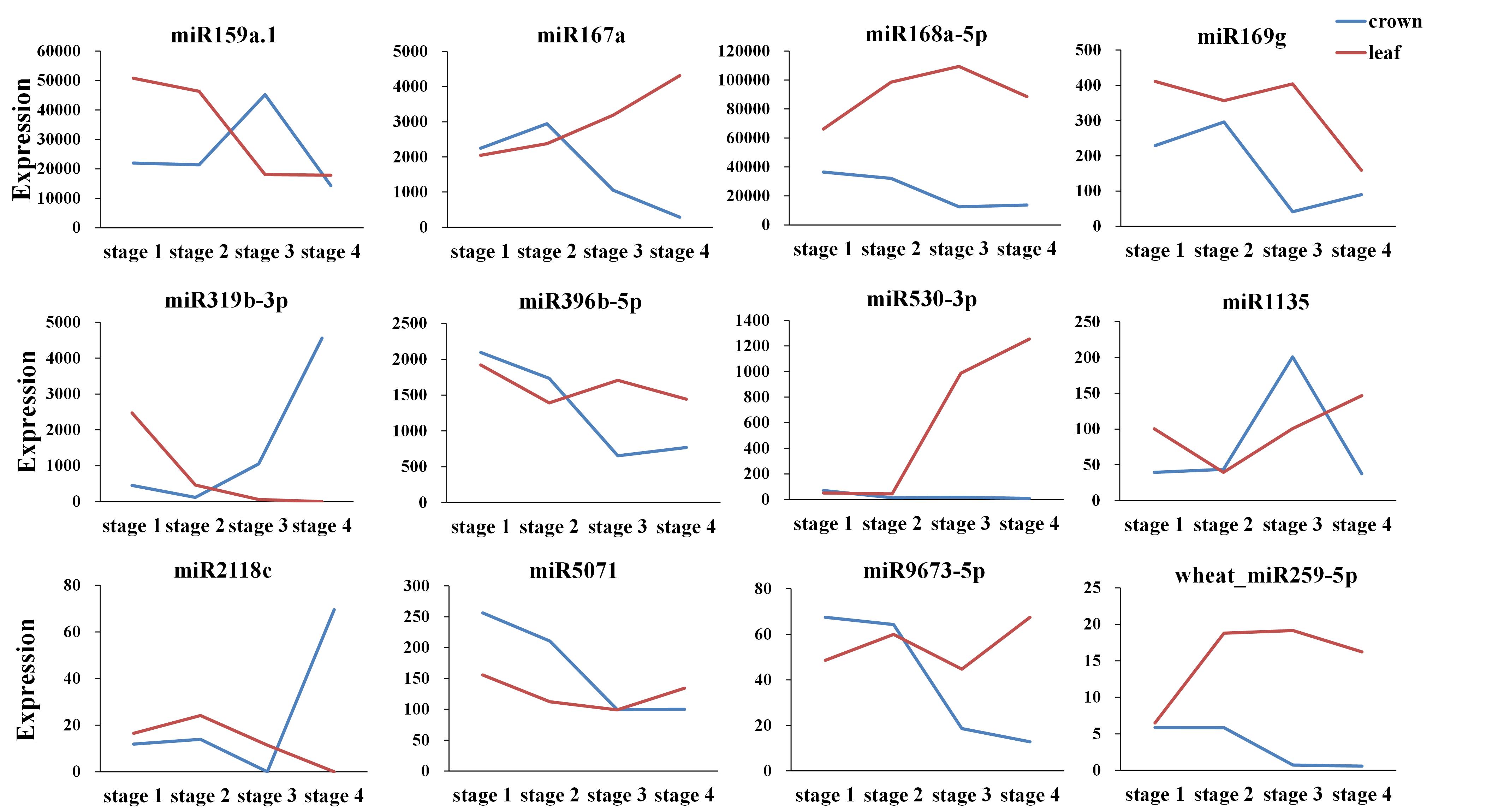

Supplement: Supplementary file 7 — Additional file 7: Figure S3. miRNAs with different expression patterns in crown and leaf tissues at the winter dormancy stage, spring green-up stage and jointing stage. [file 12864_2021_8048_MOESM7_ESM.jpg]
